# Controlling Growth of Poly (Triethylene Glycol Acrylate-*Co*-Spiropyran Acrylate) Copolymer Liquid Films on a Hydrophilic Surface by Light and Temperature

**DOI:** 10.3390/polym13101633

**Published:** 2021-05-18

**Authors:** Aziz Ben-Miled, Afshin Nabiyan, Katrin Wondraczek, Felix H. Schacher, Lothar Wondraczek

**Affiliations:** 1 Otto Schott Institute of Materials Research (OSIM), Friedrich Schiller University Jena, D-07743 Jena, Germany; aziz.ben.miled@uni-jena.de; 2 Institute of Organic Chemistry and Macromolecular Chemistry (IOMC), Friedrich Schiller University Jena, D-07743 Jena, Germany; afshin.nabiyan@uni-jena.de (A.N.); felix.schacher@uni-jena.de (F.H.S.); 3 Leibniz Institute of Photonic Technology (Leibniz IPHT), D-07745 Jena, Germany; katrin.wondraczek@leibniz-ipht.de; 4 Jena Center for Soft Matter (JCSM), Friedrich Schiller University Jena, D-07743 Jena, Germany

**Keywords:** dual-stimuli-responsive materials, thin films, out-of-equilibrium

## Abstract

A quartz crystal microbalance with dissipation monitoring (QCM-D) was employed for in situ investigations of the effect of temperature and light on the conformational changes of a poly (triethylene glycol acrylate-*co*-spiropyran acrylate) (P (TEGA-*co*-SPA)) copolymer containing 12–14% of spiropyran at the silica–water interface. By monitoring shifts in resonance frequency and in acoustic dissipation as a function of temperature and illumination conditions, we investigated the evolution of viscoelastic properties of the P (TEGA-*co*-SPA)-rich wetting layer growing on the sensor, from which we deduced the characteristic coil-to-globule transition temperature, corresponding to the lower critical solution temperature (LCST) of the PTEGA part. We show that the coil-to-globule transition of the adsorbed copolymer being exposed to visible or UV light shifts to lower LCST as compared to the bulk solution: the transition temperature determined acoustically on the surface is 4 to 8 K lower than the cloud point temperature reported by UV/VIS spectroscopy in aqueous solution. We attribute our findings to non-equilibrium effects caused by confinement of the copolymer chains on the surface. Thermal stimuli and light can be used to manipulate the film formation process and the film’s conformational state, which affects its subsequent response behavior.

## 1. Introduction

Controlling and understanding polymer adsorption at solid–liquid interfaces is of key importance in, e.g., coating [1], lubrication [2], surface adhesion [3], or colloid stabilization [4]. Polymer adsorption on a surface may occur in two general ways: by chemisorption or by physisorption. Chemisorption happens when polymers attach to a solid surface through a covalent bond. This type of adsorption is irreversible, and it is employed in many applications, such as repellant surface layers [5] or other types of functional coatings [6]. Alternatively, physisorption takes place as a result of physical attractive forces between polymer segments and the surface [7]. Physisorbed chains may consist of loops, tails and trains [8]. In general, physisorption of polymers from a bulk liquid on a solid surface can be either irreversible or reversible [9]. Irreversibility is usually achieved using hydrogen bonding or other dipolar forces, dispersive forces, or attractions between charged groups along the polymer backbone and the surface [10]. It typically occurs on metals, semiconductors, inorganic glasses, or sol-gel layers such as polydimethylsiloxane (PDMS), for example, when surface oxygens of the substrate form strong hydrogen bonds with the polymer [11]. Similarly, various macromolecules (polymers, proteins, DNA, etc.) are prone to adsorb strongly on oxide glass surfaces through hydrogen bonds or other physical forces (electrostatic attractions, hydrophobic interactions in the solvent) [1,12,13]. On the other hand, physisorption from a solution is reversible when the polymer binds weakly to the surface and has only few conformational restrictions.

**Figure 1:**
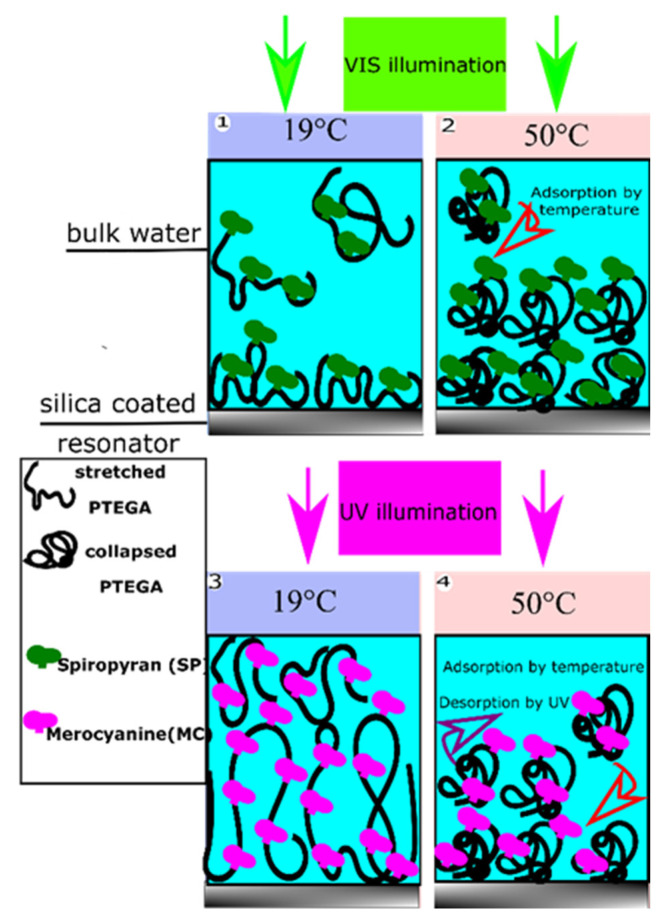
Coil to globule transition at different temperatures shown by way of example for the dual light and temperature responsive P (TEGA-co-SP/MC) copolymer during in situ observation of adsorption and film formation on a silica surface by QCM-D.

In order to tailor surface adhesion, stimuli responsive polymers have attracted great attention in the last decades due to their ability to respond to external triggers, including temperature [14], light [15], pH [16], ionic strength [17], or combinations of thereof [18,19]. Layers formed from such polymers are expected to enable switchable surfaces which may change their properties in controllable and programmable ways [20]. Sometimes, such switching can be through combinations of multiple stimuli (e.g., light and temperature, [21]), what enables logic gate operations. Thermo-responsive polymers are the most studied stimulus responsive materials. In aqueous solution, they usually display a fully reversible hydrophilic–hydrophobic transition (Figure 1), characterized by a lower critical solution temperature (LCST) [22]. Below the LCST, the polymer swells with a random coil conformation, while above LCST, the polymer collapses into a globular state and undergoes a liquid–liquid phase separation. This transformation from coil to globule is based on hydrogen bonds that are present between the polymer chains and the surrounding water molecules at temperatures below the LCST [23]. At higher temperatures, the hydrogen bonds become weaker, leading to the dehydration of the polymer chains. Some prominent examples for this behavior are microgels [24,25], poly (*N*-isopropyl acrylamide, PNIPAM [23,26], acrylamides [27,28], poly (2-oxazolines) [29,30], poly (propylene glycol) [31,32], and poly (oligo (ethylene glycol) acrylates [33,34,35].

**Figure 2:**
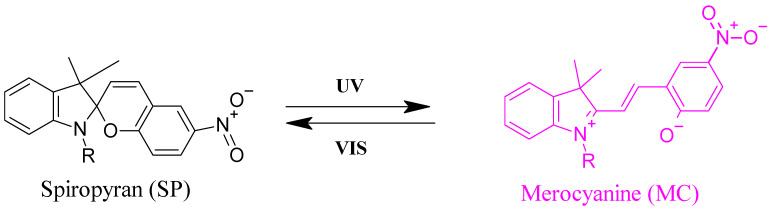
Photo switching between the spiropyran (SP) (left) and merocyanine (MC) form (right).

Incorporation of photochromic moieties into thermosensitive polymer backbones is a practical way to control their solubility in aqueous solutions by changing the temperature at which the phase transition happens through an optical stimulus [36]. Organic photochromic compounds that can be used for this purpose include spiropyran (SP) [37], azobenzene [38], and diarylethene [39]. These compounds are responsive to light irradiation through reversible or irreversible isomerization between two states of variable polarity. Isomerization reactions can be detected through observation of color changes due to photon absorption. [40] In case of SP-MC, the deep purple color of a liquid thin film upon UV irradiation originates from the absorption of UV photons causing a breakage of the C-O spiro bond in an excited singlet state, see example shown in Figure 2.

SP is one of the chromophores that is not only a photo-switch but also responds to other stimuli such as temperature, solvent, metal ions, and pH [41]. In response to UV light (λ = 365 nm), the closed nonpolar and colorless spiro form “SP” is transformed into the open, polar, colored and zwitterionic merocyanine form ”MC”. Irradiation with visible light (λ = 550 nm) causes ring closure and return to the initial state. The UV-light induced reversible isomerization of SP between nonpolar and polar states can be used to tune the phase separation of thermo-responsive polymers since the (UV-induced) polarity change affects the interaction between the polymer and the solvent. By combination with different types of backbone polymers this enabled, e.g., controlled foaming or bubble formation using light irradiation of spiropyran sulfonate surfactants [42], rewritable optical storage in spiropyran-doped liquid crystal polymer film [43], or controlling the enzymatic activity on orthogonally functionalized glycidyl methacrylate with spiropyran [44]. SP-incorporating poly (oligo (ethylene glycol) acrylate)-based copolymers have been synthesized by nitroxide mediated polymerization with varying amounts of SP (from 0 to 16 mol%). The visible light irradiation of the copolymer dissolved in pH 8 TRIS buffer resulted in a decrease in its cloud point temperature by 30 K at 16 mol% SP content, as previously detected by UV/Vis spectroscopy [45].

A quartz crystal microbalance with dissipation monitoring (QCM-D) is a highly sensitive technique for characterizing adsorption and desorption phenomena at the solid–liquid interface. Numerous experimental investigations and modeling studies have been carried out on the viscoelastic properties of adsorbed polymer films and their solid–liquid interfacial properties using QCM-D [46,47,48]. As an exemplary case, the adsorption of PNIPAAm on modified gold and silica surfaces was studied due to its conveniently accessible LCST of ~32 °C, and also for its potential relevance in biomedical applications [49]. These studies showed different behaviors of the adsorbed polymer depending on its state of adsorption, e.g., whether chemisorbed [50,51] or physisorbed [52,53]. The adsorption of thermosensitive block copolymers based on PNIPAAm on a gold surface was also investigated by QCM-D [54]. For example, the adsorption mechanism of a pentablock terpolymer poly (*N*-isopropylacrylamide)_x_-*block*-poly (ethylene oxide)_20_-*block*-poly (propylene oxide)_70_-*block*-poly (ethylene oxide)_20_-*block*-poly (*N*-isopropylacrylamide)_x_ (PNIPAAm_x_-*b*-PEO_20_-*b*-PPO_70_-*b*-PEO_20_-*b*-PNIPAAm_x_) on gold was found to be affected by several parameters including concentration, relative block length, temperature, and the substrate’s physical properties. Furthermore, adsorption properties of pH sensitive cationic polyelectrolytes, e.g., poly (diallyl dimethyl ammonium chloride) or poly (allyl amine hydro-chloride) (PAH) on gold and silica surfaces were studied using QCM-D [55]. It was found that the adsorption property of the polyelectrolyte depends on the solid surface, solution concentration, and solution pH. As another example, QCM-D was employed to study the adsorption of polyelectrolyte monolayers of anionic poly (styrene sulfonate) (PSS) on amino-functionalized silica, as well as cationic PAH and poly-L-lysine (PLL) on bare silica [12]. In this example, the thickness of the polyelectrolyte monolayers increased when increasing the ionic strength (salt concentration) and the polyelectrolyte concentration.

Interestingly, also the light-induced swelling behavior of spin-coated thin layers of P (NIPAM-*co*-SPA) copolymers was described on the basis of QCM-D investigations [56]. However, although the employed deposition method is technologically important for the fabrication of thin films on solid surfaces, it also has the limitation of making the film prone to delamination once the solvent wets the substrate [57]. Nevertheless, studying adsorption of such copolymers appears very interesting from a physical point of view; they can adopt different conformations, which can be tuned by light irradiation and temperature.

In this paper, we report on the conformational change of the dual light and temperature responsive copolymer P (TEGA-*co*-SPA) in solution and confined at the silica-water interface using QCM-D measurements. We monitor the simultaneous effect of UV light irradiation and temperature changes on the co-polymer’s adsorption behavior. Optical irradiation of the copolymer solution while undergoing adsorption provided us with direct access to the question as to how light can be used to tailor the kinetics of film formation and film conformation below and above the LCST.

## 2. Materials and Methods

### 2.1. Materials

P (TEGA-*co*-SPA) synthesis was reproduced from reference [45]. More details are provided in the Supplementary Section. Using this method, spiropyran acrylate (SPA) was obtained as a yellow powder. Commercial TEGA monomer was copolymerized with 15 mol% SPA in the initial monomer mixture. The obtained copolymer was investigated via size exclusion chromatography with triple detection to obtain absolute molar masses and ^1^H liquid NMR to determine the composition by comparing the signal of the SPA moiety (8.2 ppm, 2H) and the TEGA moiety (3.3 ppm, 3H). The fraction of SPA in the obtained copolymer was between 12–14 mol%, the molar mass *M*_n_ was about 33,000 g/mol with a dispersity index PDI = 1.7. An aqueous solution of 0.15 wt.% P (TEGA-*co*-SPA) was obtained by diluting the copolymer in deionized water. Deionization was done using a Thermo Scientific Barnstead MicroPure water purification system to a resistivity of 18.2 MΩ cm^−1^.

### 2.2. Dynamic Light Scattering

DLS measurements were performed using an ALV Laser CGS3 Goniometer (ALV GmbH, Langen, Germany) equipped with an He-Ne laser (λ = 633 nm) and an ALV-7004/USB FAST correlator. All DLS measurements were performed at 25 to 77 °C. To determine the hydrodynamic radius, three measurements of 30 s each were performed at an angle of 90 ° The analysis of the obtained correlation functions was performed using the correlator software (Correlator 3.2 beta 1).

### 2.3. QCM-D Experiment

QCM-D measurements were performed using a window module mounted on the QCM sensor (Q-sense E1 Biolin Scientifc, Västra Frölunda, Sweden). The employed sapphire window had an optical transmittance of >80% in the wavelength range 300 to 400 nm, in which UV irradiation was conducted.

AT-cut quartz crystal sensors coated with a 50 nm silicon dioxide layer (fundamental resonance frequency of typically ~4.95 MHz, sensor area 1.54 cm^2^) were purchased from Biolin Scientific, Sweden. Prior to experiments, the quartz sensor was cleaned by soaking in a 2 vol% sodium dodecyl sulfate SDS solution for 30 min, rinsing with ultra-pure water, blow-drying with a gentle nitrogen flow and, finally, exposing to a UV/ozone cleaner for 15 min.

Several overtones were acquired, although the third overtone was generally selected for further analysis because of its level of energy trapping at this particular overtone when operated in liquids [58].

For studying the dual light and temperature induced conformational response of the P (TEGA-*co*-SPA) solutions, all experiments were performed in the liquid exchange mode by first purging with ultra-pure water for 30 min at 19 °C at a flow rate of 50 µL/min. To avoid the formation of bubbles that can oscillate or migrate over the quartz crystal surface, all solutions were degassed in an ultrasonic bath (Elmasonic S 80) for 10 min prior to injection. If not otherwise stated, irradiation of the sensor with the light source was started 20 min after equilibration and referencing under continuous water flow was completed. The diluted P (TEGA-*co*-SPA) aqueous solution was then introduced into the chamber at 30 min and at a temperature of 20 °C ± 0.02 °C. At this point, the flow rate was reduced to 20 µL/min. Temperature ramping was conducted from the starting temperature of 20 °C up to a maximum of 47 °C, applying a constant heating rate of 0.2 K/min.

In the isothermal irradiation study, the P (TEGA-*co*-SPA) aqueous solution was fed for 25 min through the window module at a constant temperature prior to irradiation.

Irradiation was done with a fluorescent lamp (visible light) or using an ultraviolet spotlight (365 nm, Opsytech, Ettlingen, Germany). The power of UV LED was fixed at 10% via an LED controller (with a maximum nominal power density of 25 W/cm^2^); the sample-to-LED distance was maintained at 75 mm.

During each run, changes in the resonance curves of the third overtone were continuously monitored and evaluated. The two resonance parameters under investigation were the change in dissipation factor Δ*D*_3_, and the shift in resonance frequency Δ*f*_3_/3 being related to the mass of the adsorbate and the dynamically coupled liquid. While the resonance frequency shift Δ*f*_3_/3 is more sensitive to the mass of the film, the variation of the dissipation factor Δ*D*_3_ is related to viscous losses and interfacial sliding [47]. The acquired datasets were corrected for each sensor using a temperature sweep in pure water for reference, see also Supplementary Material (Figure S2 and Table S1). This temperature correction was carried out by subtracting the calibration curve (pure water on sensor) from the one obtained in the presence of the dissolved copolymer. Furthermore, irradiation of the quartz crystal with UV light induced an increase in Δ*f*_3_/3 by a few Hz. This behavior was previously attributed to photo-induced mechanical stress [59,60]. A further calibration was, therefore, done for UV-illumination by subtracting the effect of the UV light on the crystal for the non-isothermal measurements, see calibration curve in Supplementary Material (Figure S3).

### 2.4. Data Evaluation

QCM-D is an established, sensitive tool to study in situ the adsorption from a liquid in contact with the surface of a quartz crystal resonator [61,62,63]. The resonance frequency is defined as the frequency where the electrical conductance of the equivalent circuit is maximal. If a Lorentzian peak function is fitted to the conductance curve, two parameters are obtained describing the complex resonance frequency $f_{n}^{*}$, the resonance frequency *f_n_* of the quartz as the real part and the half width at half maximum of the resonance peak, $\Gamma_{n}$ representing the imaginary part. A thin layer or any loading on the quartz crystal surface generates a complex resonance frequency shift Δ$f_{n}^{*}$ compared to the empty state, which can again be separated into $\Delta f_{n}$ (the real part) and ${\Delta \Gamma }_{n}$ (the imaginary part),

(1) $$\Delta f_{n}^{*}=\Delta f_{n}+i\Delta \Gamma_{n}$$

The fundamental resonance frequency of AT cut quartz crystal resonators operated in shear mode is typically near 5 MHz. More resonances are observed at the odd harmonics of this fundamental frequency, where the subscript *n* refers to the *n*th harmonic (i.e., *n* = 1 for the fundamental resonance frequency of 5 MHz, and *n* = 3 for the third overtone partial at ~15 MHz). The adsorbed rigid mass can be quantified using the Sauerbrey equation [64], where the adsorbed areal mass density *m_f_* correlates with $\Delta f_{n}^{*}$ [58].

(2) $$\frac{\Delta f_{n}^{*}}{f_{1}}=\frac{-2\;f}{Z_{q}}\;m_{f}$$

where *f*_1_ is the fundamental frequency, *f* is the measured resonance frequency and *Z_q_* = 8.8 × 10^6^ kg^.^m^−2.^s^−1^ is the acoustic impedance of quartz. The Sauerbrey equation is strictly valid only for rigid films. For a viscoelastic film immersed in liquid environment, a viscoelastic correction is required to account for viscous dissipation, whereby softness reduces the apparent rigid Sauerbrey thickness [58],

(3) $$\frac{\Delta f_{n}^{*}}{f_{1}}=-\frac{\;\omega \;m_{f}}{\pi Z_{q}}\left(1-\frac{Z_{liq}^{2}}{Z_{film}^{2}}\right)$$

where *ω* = 2*πf*, $Z_{liq}$ = $\sqrt{n2\pi if_{1}\rho_{liq}\eta_{liq}}$, $Z_{film}$ = $\sqrt{\left(\rho_{film}G_{film}\right)}$; $Z_{liq}\;$is the acoustic field impedance of the liquid, $Z_{film}$ the acoustic field impedance of the film, $\rho_{liq}\;$is the density of the liquid, $\eta_{liq}$ the dynamic viscosity of the liquid, $\rho_{film}$ the density of the film and $G_{film}$ the shear modulus of the film.

Aside mass or Sauerbrey thickness, QCM-D simultaneously monitors dissipation which can be expressed by the factor *D*,

(4) $$D_{n}=\frac{2\;\Gamma_{n}}{f_{n}}$$

Viscoelasticity, but also further effects such as surface roughness cause a shift $\Delta D_{n}$ of the dissipation factor [65,66,67]. When the crystal is immersed in a Newtonian liquid [68], the resonance frequency and dissipation factor shifts are proportional to the square root of liquid density $\rho_{liq}$ times the liquid dynamic viscosity $\eta_{liq}\;$according to Kanazawa–Gordon–Mason relation [69],

(5) $$\frac{\Delta f_{n}}{f_{1}}=\frac{-1}{\pi Z_{q}}\sqrt{\omega \;\rho_{liq}\;\eta_{liq}}$$

(6) $$\Delta D_{n}=\frac{2}{n\pi Z_{q}}\sqrt{\omega \;\rho_{liq}\;\eta_{liq}}$$

## 3. Results and Discussions

### 3.1. Phase Separation of P (TEGA-Co-SPA) in Dilute Aqueous Solution

**Figure 3:**
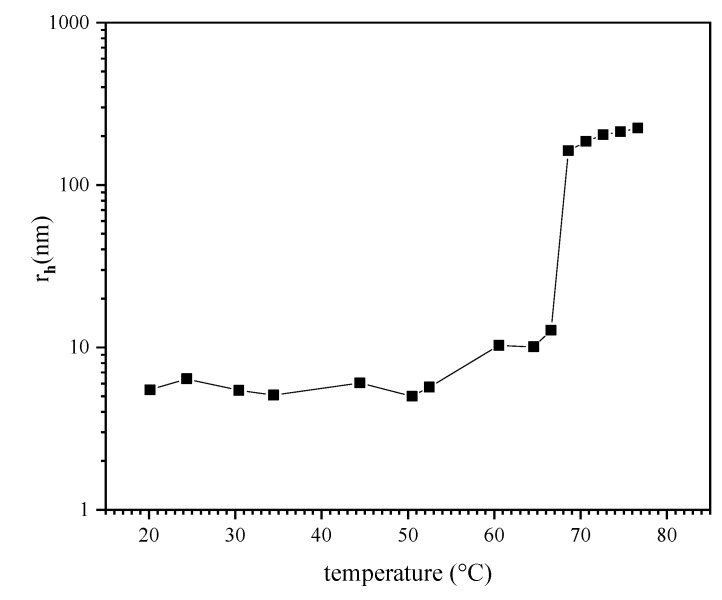
Hydrodynamic radius of a P (TEGA-co-SPA) copolymer in aqueous solution upon heating as determined from DLS measurements.

DLS data shown in Figure 3 provide an initial view at the effect of temperature on aggregation in the P (TEGA-co-SPA) polymer solutions containing between 12 and 14 mol% of spiropyran in terms of the hydrodynamic radius. In order to reduce the effect of particle aggregation, we chose to work with a dilute concentration of 0.06 wt.% (optically clear at room temperature). This is below the concentration used for DLS studies of similar thermoresponsive copolymers [70]. The hydrodynamic radius observed by DLS shows a sudden transition at a temperature of ~66 °C. Below this temperature, the polymer chains exist as individually dissolved polymer chains with small hydrodynamic radius of approximately 4–6 nm. Above 66 °C, aggregates (mesoglobules) with larger hydrodynamic radius of around 100–200 nm are formed. These values are comparable in size to other known polymers with a LCST [71,72]. At temperatures below the LCST, the copolymer chains are well solvated through hydrogen bonds [73,74]. Above the LCST, these exhibit van der Waals character, e.g., such as reported for PNIPAAm [70,75]. Interestingly, the observed transition temperature occurs ~23 K above the reported cloud point for the same copolymer composition diluted in pH 8 TRIS buffer, as detected by UV/VIS spectroscopy [45]. This observation is attributed to the effect of salts contained in the buffer on the electrostatic interactions between the copolymer and water as reported recently for various thermoresponsive polymers [76].

### 3.2. Effect of UV-Irradiation on the Hydration of P (TEGA-Co-SPA) Films below and above the LCST

**Figure 4:**
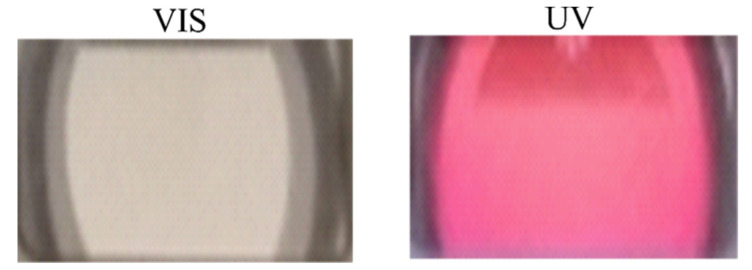
Effect of illumination on the P (TEGA-co-SPA) liquid film color. The photos were taken by a normal camera on the top of the QCM-D window cell.

The P (TEGA-co-SPA) liquid thin film adsorbed onto silica appears almost transparent under visible light, but switches to deep purple upon UV irradiation (Figure 4). As explained before, the deep purple color of the liquid thin film upon UV irradiation originates from the absorption of the UV photons causing a breakage of C-O spiro bonds in an excited singlet state yielding the colored MC form. Due to the physisorption of the copolymer in our case the chains of MC are enforced to rearrange in a way the ethylene oxide groups point to the solution that may stabilize the merocyanine form via hydrogen bonds.

**Figure 5:**
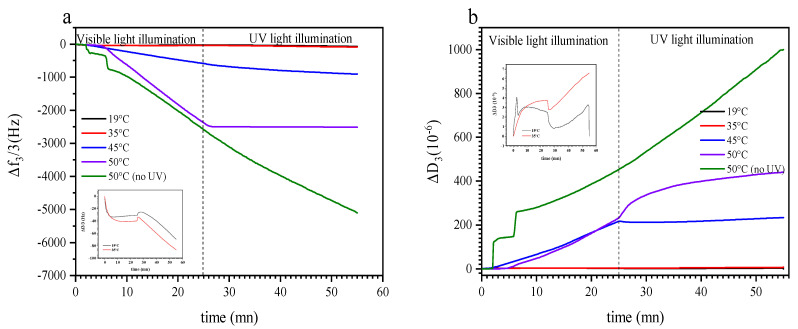
Variation of Δ*f*_3_/3 (**a**) and Δ*D*_3_ (**b**) versus time of PTEGA-co-SPA at the interface silica-water at a constant temperature. The inset is a zoom at Δ*f*_3_/3 in the range of 19 °C to 35 °C.

By way of example, we selected different temperatures for isothermal treatment with and without illumination below and above the LCST when investigating with QCM-D. Figure 5a shows the effect of switching from visible to UV light irradiation on Δ*f*_3_/3 as a function of time at 19 °C, 35 °C, 45 °C, 50 °C; and 50 °C when the sensor was not irradiated with UV light, respectively. At 19 °C and 35 °C, the introduction of the copolymeric solution inside the window cell causes an initial frequency decrease (mass increase) followed by a slower frequency decrease as the system saturates at −31 Hz and −40 Hz, respectively. Starting at 25 min, the sensor surface was irradiated with UV light, what caused a marginal increase in Δ*f*_3_/3 of a few Hz, followed by a linear decrease in the frequency in the next several minutes, see inset of Figure 5a. In comparison, when there is no light switch at 19 °C, Δ*f*_3_/3 and Δ*D*_3_ signals do not show any significative change, see supporting information (Figure S5a,c). The spike of Δ*f*_3_/3 occurring immediately after illumination attributed to the effect of UV light on the crystal as described in the Materials and Methods section. The shallow linear decrease in the frequency shift is probably due to an increase in acoustic thickness as the copolymer chains swell. A similar result was observed in a previous study [56], where PNIPAAm-co-SPA thin films were illuminated with a UV lamp at 19 °C. In this material, the behavior was explained by a photoinduced hydration due to the photoisomerization of the rather hydrophobic spiropyran into the distinctly more hydrophilic merocyanine when the thermo-responsive part of the copolymer is sufficiently hydrophilic.

At 45 °C and 50 °C, Δ*f*_3_/3 decreases linearly once the copolymer solution is in contact with the sensor. This decrease in Δ*f*_3_/3 is high in magnitude, reaching 0.58 and 2.38 kHz, respectively, after 25 min of continuous solution feed and visible light irradiation. Interestingly, UV light illumination affects Δ*f*_3_/3 differently at 45 °C and 50 °C. Although at 45 °C the rate of the observed decrease in Δ*f*_3_/3 slows down and causes a deviation from linearity, it stabilizes at a constant (but very low) value at 50 °C. Noteworthy, when continuing visible illumination and turning UV off beyond 25 min, the observed strong decrease in Δ*f*_3_/3 continues unaffected, indicating that indeed UV illumination (versus, e.g., some saturation effect) plays a role in the reaction observed at 50 °C (see also Figure S5b,d). We attribute this observation to a competition between PTEGA globule adsorption on the sensor surface and photoconversion of spiropyran to merocyanine. When there is no UV irradiation, surface adsorption is facilitated and the observed Sauerbrey thickness increases during prolonged solution injection. This process is interrupted by the conversion of the unipolar spiropyran to the polar merocyanine, which enhances the stability of the solution and thereby reduces the adsorption rate. Similar observation have been made for azobenzene surfactant adsorption and desorption at the air–water interface under UV irradiation [77].

Figure 5b shows the evolution of Δ*D*_3_ corresponding to Figure 5a. At 19 °C and 35 °C, Δ*D*_3_ shows low values in the first 25 min, suggesting that the film is forming a monolayer at the silica surface. Once the surface is irradiated with UV light, Δ*D*_3_ increases linearly at both temperatures and reaches ~3 × 10^−6^ and 6 × 10^−6^, respectively, at 19 °C and 35 °C after around 55 min. At the higher temperatures of 45 °C and 50 °C, Δ*D*_3_ increases similarly (although at much higher rate) for as long as the sensor is irradiated with visible light. Once UV illumination is switched on at these temperatures, there is a very significant effect on dissipation: at 45 °C, Δ*D*_3_ decreases slightly and subsequently reaches a plateau, while at 50 °C, Δ*D*_3_ apparently evolves in a square root dependence on time, which could indicate some kind of diffusive process. Interestingly, the latter extends far beyond the time at which surface adsorption is interrupted (Figure 5a); we note that dissipation evolves as a convolution of swelling effects within the film, as well as adsorption from the solution, which are both affected by the two stimuli of temperature and light. When adsorption stops, conformational changes can still proceed within the film, but these would be significantly slower in their response rate due to the reduced film mobility as compared to the polymer in solution. The observed square root dependence on time corroborates this interpretation.

### 3.3. Dual Temperature and Light Effect on the Build-Up of P (TEGA-co-SPA) Layers on Silica Surfaces

Temperature ramping was carried out in order to investigate the concomitant effect of temperature and light on the conformational change of the P (TEGA-co-SPA) diluted solution during adsorption. We started by analyzing the behavior of a P (TEGA-co-SPA) thin film being formed on the QCM-D sensor surface.

**Figure 6:**
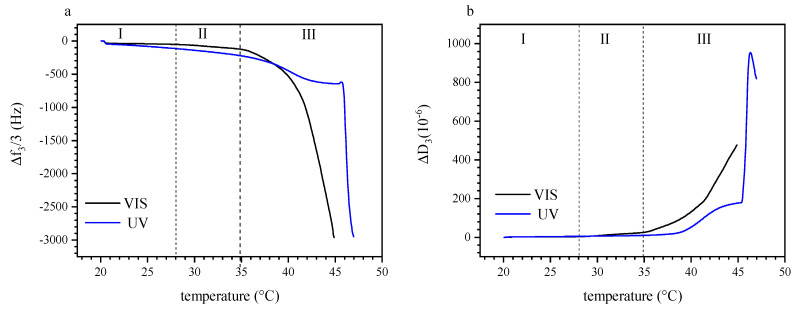
(**a**) Variation of normalized Δ*f*_3_/3 as a function of temperature upon irradiation of P (TEGA-co-SPA) copolymers at the silica–water interface, the copolymer was introduced at T = 20 °C, (**b**) variation of Δ*D*_3_ as function of temperature of the same solution. Blue curves: Upon UV illumination, Black curves, upon illumination with visible light. The labels (I–III) mark the three regimes of adsorption and film response discussed in the text.

Figure 6a shows the variation of the normalized resonance frequency shift Δ*f*_3_/3 over a temperature range of 20 °C to 47 °C, comparing the effects of visible light irradiation and UV irradiation (365 nm). Under UV exposure, we observe an initial, slow decrease in Δ*f*_3_/3 between 21 °C and 28 °C, which is less pronounced under visible light. This difference suggests that the sensed mass (load) increased with UV irradiation, which could be attributed to additional hydrodynamically coupled water inside the adsorbed film in this temperature range. Any masses as retrieved by QCM-D are non-specific, that is, both polymer and water (or solvent in general) bound in the adsorbed films are detected. For instance, in case of protein adsorption, an additional molecular weight increase of ~30% was reported, that was attributed to water bound to a protein molecule in solution [78]. In our present case, we believe that the photoisomerization of the spiropyran with UV irradiation results in a higher trapped amount of water inside the layer of P (TEGA-co-SPA) when it is sufficiently hydrophilic [56]. For visible light irradiation, we note a change in the slope of Δ*f*_3_/3 over T at ~28 °C; under UV irradiation, such a change is not observed until a much higher temperature of near ~47 °C. We attribute this change of the slope to a sudden increase in the amount of the adsorbed copolymer chains at the sensor surface. As we are approaching the LCST, one should expect that the copolymer is gradually collapsing and releasing water. This dehydration should express as increased Δ*f*_3_/3 values as reported, e.g., for PNIPAAm layers adsorbed on a hydrophobic gold surface [52].

However, we must note again that we do not observe the properties of a preexisting film, but the process of a film being formed in situ from a photo-thermoresponsive solution. Thus, we argue that the observed decrease in Δ*f*_3_/3 (despite water release) is a result of polymer adsorption and film growth, which dominates over any water release reaction, in particular, as the hydrophilic coil to hydrophobic globule transition occurs already in solution, and only to a smaller extent within the film. At 35 °C, Δ*f*_3_/3 of the visible light irradiated sensor decreases drastically which we relate to the liquid–liquid phase separation. Interestingly, this large decrease in Δ*f*_3_/3 occurs at about 4–8 K lower than the reported cloud point temperature of the same copolymer in TRIS buffer solution when irradiated with 540 nm visible light [45]. This difference between the cloud point temperature detected by UV/Vis spectroscopy and the phase transition temperature determined acoustically on a surface suggests that the confinement affects the coil-to-globule transition of the copolymer at the interface. At 38.3 °C, we note a change in the the feature of Δ*f*_3_/3 over T of the UV irradiated solution, with an initial acceleration (higher negative slope) of adsorption, followed be a deceleration and a plateau reaching up to ~45 °C. This is in line with our isothermal observations summarized in Figure 5a, where UV irradiation at higher temperature decelerates film adsorption up to a certain extent. Here, the deceleration sets in just before LCST as would be occurring under visible illumination. At 45.7 °C, we observe a sudden, strong acceleration of the adsorption rate, with a sharp decrease in Δ*f*_3_/3. This is attributed to the retarded P (TEGA-co-SPA) LCST in the aqueous solution, shifted to a higher temperature due to the increase in the hydrophilicity of the polymer as induced by the 365 nm UV light irradiation. A similar temperature shift was also observed for the bulk material using UV-Vis spectroscopy, although the transition temperature happening at the interface silica-water was lowered by 2–3 K [45].

The dissipation data corresponding to the observed cases of Δ*f*_3_/3 is displayed in Figure 6b. Here, too, we distinguish the three regions of (I) T < 28 °C, (II) 28 °C < T < 35 °C, and (III) T > 35 °C (marked as I–III in Figure 6). Again, an increase in dissipation correlates to the enhancement of coupling between water molecules and polymer chains due to the photo-induced hydration under UV illumination (region I). Moreover, there is a significant difference of the sensed masses on the sensor, depending on the type of irradiation. In the temperature range of 21 °C to 28 °C, Δ*D*_3_ increases by a factor of about two, that is, from 1.6 × 10^−6^ to 3.3 × 10^−6^ and from 2.2 × 10^−6^ to 4.9 × 10^−6^ for the visible and UV irradiated film, respectively. In the temperature range of 28 °C to 35 °C, Δ*D*_3_ increases more strongly for the solution exposed to visible light as compared to the one irradiated with UV light. This observation corroborates our interpretation that the competition between dehydration and adsorption starts already several K below the commonly reported LCST. At 35 °C, Δ*D*_3_ increases dramatically, in agreement with the resonance frequency data. According to the change of slope at ~38 °C, the dehydration happens gradually also under UV light. In this case, the retarded phase transition reflects in the over damping of the layer happening at ~46 °C, where the magnitude of Δ*D*_3_ reaches 953 × 10^−6^.

**Figure 7:**
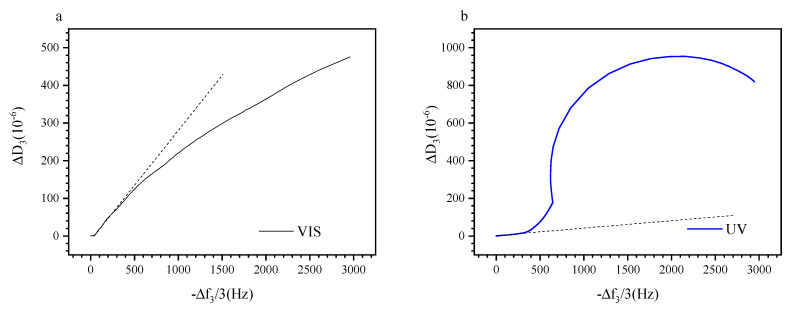
(**a**) Variation of Δ*D*_3_ as function of −Δ*f*_3_/3 when the sensor is continuously exposed to visible light, (**b**) variation of Δ*D*_3_ as function of −Δ*f*_3_/3 when the sensor is irradiated with UV light. Blue curves: upon UV illumination, black curves: upon visible illumination.

Examining the change of the energy dissipation as function of the negative frequency shift allows to eliminate the temperature as a variable and to focus on the effect of the light irradiation on the viscoelastic properties during layer build-up [79]. Figure 7a,b show the evolution of Δ*D*_3_ as function of −Δ*f*_3_/3, respectively, for visible and UV irradiated P (TEGA-co-SPA) at the silica–water interface. Interestingly, both properties are not directly proportional; furthermore, the adsorbed film does not evolve in the same way whether it is irradiated with visible or UV light. Under Vis illumination, the change in dissipation underrepresents the change in resonance frequency whereas under UV light, it strongly exceeds the frequency change. A linear correlation between both properties is found only in the onset region of film formation, i.e., within 0 to 550 Hz (Vis) and 0–320 Hz (UV), where surface coverage of the layer is still low. In this range, the hydrodynamic thickness is expected to be small, and the number of polymer molecules adsorbed physically through trains, loops, and tails is negligible [80]. For as long as the dissipation values are low and Δ*D*_3_ increases linearly with −Δ*f*_3_/3, we assume that the viscoelastic properties of the film remain unchanged and the parameter variations are solely due to continuous adsorption. The occurrence of such a region was similarly observed by QCM-D for different adsorbing systems, including polyelectrolytes [81] and homopolymers on gold [52].

Beyond the linear onset regime, there are pronounced effects of temperature and illumination. For the visibly irradiated surface, we observe a decrease in dissipation as the coverage of the surface is increasing. This evolution can be explained by the densification of the film once the surface is saturated. For the UV irradiated layer, we observe a strong excess in dissipation which saturates at about 2000 Hz. The spiral shape is similar to previous observations made on polystyrene brushes in cyclohexane [82]. It indicates that the deposited film more pronouncedly interacts with the bulk solution, resulting in enhanced dissipation when the copolymer is in its polar (MC) state. In this case, we should expect a film with lower density as UV light leads to decelerated absorption and a polarity change, therefore the adsorbate has less time to rearrange itself as one its only irradiated with visible light.

The LCST of dilute P (TEGA-*co*-SPA) depends on illumination conditions. Adsorption kinetics and film growth at the silica–water interface can, therefore, be controlled through temperature and illumination conditions, relying on the thermally induced transition from hydrophilic coil arrangement to hydrophobic globules of the PTEGA components, and on the transition in polarity of the SPA-MC component controlled through illumination. Similarly, the film itself responds to thermal as well as optical stimuli through variable dissipation of acoustic excitation. Our results show that the coil-to-globule transition temperature is lower in diluted samples exposed to an adsorbing surface as compared to the solution. The conformational state of the adsorbed polymer chains is controlled by surface confinement and kinetics, whereby non-equilibrium conformational states could be frozen in for long times after adsorption [83]. A present assumption is that non-equilibrium effects originate from the polymer density and conformation at the interface of the adsorbing surface and the surrounding polymer solution or melt above its glass transition [84], which are kinetically frozen-in as a result of adsorption. For example, the slow rejuvenation of compressed polyethylene oxide PEO adsorbed on mica was found to be caused by the low mobility of the polymer chains in their adsorbed state [85]. Glassy dynamics of thermoresponsive, adsorbed polymers were investigated on solid substrates, e.g., latex particles in water. When the temperature was raised above the LCST temperature, PNIPAAm underwent a conformational transition from adsorbed loops to globules [86]. This transition process was slow: the relaxation time was found to vary between a few hundred to several thousand minutes [87]. Notwithstanding the difference in chemical structure between PNIPAAm and PTEGA, we assume that the difference between the bulk and surface LCST temperatures found here for P (TEGA-*co*-SPA) is likely due to similar kinetic considerations.

Another interesting aspect of the non-equilibrium nature of the adsorbed layer is related to the interplay between adsorption and wetting. The evolution of the frequency and dissipation shift as functions of temperature illustrate experimentally the surface-driven phase separation in polymer solutions, as predicted by Cahn [88]. Water and the P (TEGA-*co*-SPA) copolymer form one single solution phase at low temperatures. When the temperature of the system is increasing and, at the same time, the interaction between the solvent and the polymer is varied through an optical stimulus [89], we expect the system to first approach the wetting point at which the mixed and the de-mixed state of the binary mixture coexist. A further increase in temperature results in phase separation. Thereby, the phase with lower interfacial energy wets the silica surface [90]. Our QCM data supports this hypothesis, similar to previous observations on the adsorption of PNIPAAm on hydrophobic gold surfaces [52].

Although it is often claimed that thin hydrogel films are hydrophobic above their LCST, we show that SPA-copolymerization provides a means to circumvent this issue. For example [56], UV light exposure was found to not affect the hydration of PNIPAAm containing 2.5 mol% SPA when the temperature was above the LCST. This was explained by confinement of the chromophore within isopropyl groups, and the hydrophobic backbone of PNIPAAm. In our case, we found that UV light decelerated the growth of the wetting layer at 45 °C and 50 °C due to a competition between the copolymer globule adsorption and photoconversion of spiropyran to merocyanine facilitating desorption (Figure 1). In the absence of UV irradiation, the copolymer escapes from the solvent toward the silica surface, and thickness of the wetting layer increases for as long as the feeding solution is continuously injected. However, when illuminating with UV light, spiropyran rapidly converts to the polar merocyanine, leading to layer swelling and, eventually, globule desorption. The further difference between our observations and previous studies on PNIPAAm-SPA are attributed to different deposition techniques, major differences in the amount of the chromophore and even the difference in molar mass of the employed copolymer, which sets variable constraint on polymer conformation and deposition kinetics. A hydrogel film of PNIPAAm deposited by spin coating may delaminate from the surface due to osmotic stress caused by interaction with water molecules [91], even above LCST. On the other hand, P (TEGA-*co*-SPA) surface rearrange both below and above the LCST; the isopropyl groups concentrate near air or other hydrophobic phases, whereas ethylene oxide groups rather orient towards water [92].

## 4. Conclusions

The conformational change of a thermal and light responsive copolymer layer of P (TEGA-*co*-SPA) on silica surfaces was investigated using quartz crystal microbalance with dissipation monitoring (QCM-D). First, we elucidate the effect of isothermal UV light illumination on the hydration state of the liquid film below and above its LCST. Second, we show that the phase separation temperature of the confined copolymer at the interface shifts to lower temperatures, namely 4–8 K lower compared to the cloud point temperatures as reported by UV/VIS spectroscopy in dilute aqueous solution. We attribute this difference to the formation of non-equilibrium adsorbed multilayers on the silica surface. Finally, we demonstrate that the built-up wetting layer displays variation of its viscoelastic properties with temperature and illumination conditions.

## Data Availability

All datasets reported in this study are available from the corresponding author on reasonable request.

## Supplementary Materials

The following are available online at https://www.mdpi.com/article/10.3390/polym13101633/s1, Figure S1: Synthetic route for 2-(3′,3′-Dimethyl-6-nitrospiro[chromene-2,2′-indolin]-1′-yl) ethyl Acrylate (SPA); Figure S2: Characterization of P (TEGA-co-SPA) copolymer: 1H-NMR in CDCl3; Figure S3: QCM-D water background used for data correction; Figure S4: Effect of UV illumination on bare (a) and water-wet sensor (b); Figure S5: Effect of continuous visible and UV light irradiation on the resonance frequency shift Δ*f*_3_/3 (a,b) and dissipation shift Δ*D*3 (c,d) on the copolymer liquid film at 19 °C and 50 °C, respectively; Table S1: Fit parameters for the temperature correction as indicated in Figure S2.
