# Supplementary material for: Controlling Growth of Poly (Triethylene Glycol Acrylate-Co-Spiropyran Acrylate) Copolymer Liquid Films on a Hydrophilic Surface by Light and Temperature
Source: Polymers (Basel). 2021 May 18;13(10):1633. doi: 10.3390/polym13101633 (PMC8157298; doi:10.3390/polym13101633)
Supplement: Supplementary file 1 [file polymers-13-01633-s001.zip › polymers-1203306-supplementary.pdf]

Supporting Information for

Controlling Growth of Poly(triethylene glycol acrylate-*co*-spiropyran acrylate) Copolymer Liquid Films on a Hydrophilic Surface by Light and Temperature

Aziz Ben-Miled[1], Afshin Nabiyan[2], Katrin Wondraczek [3], Felix H. Schacher[2, 4], Lothar Wondraczek[1]

[1]*Otto Schott Institute of Materials Research, University of Jena, Jena, Germany*

[2] *Institute of Organic Chemistry and Macromolecular Chemistry, University of Jena, Germany*

[3] *Leibniz Institute of Photonic Technology, Jena, Germany*

[4] *Jena Center for Soft Matter (JCSM), University of Jena, Germany*

P(TEGA-*co*-SPA) copolymer synthesis

Chemicals

2-Bromoethanol (95.0%), 2,3,3-trimethyl-3*H*-indol (97.0%), 2-hydroxy-5-nitrobenzaldehyde (97.0%), and triethylene glycol acrylate (98.0%) were purchased from TCI (Zwijndrecht, Belgium) and used as received. Acryloyl chloride (97.0%) was purchased from Sigma-Aldrich (Munich, Germany) and used as received. The synthesis of the SPA monomer is described in a previous publication. All solvents were of analytical grade except 1,4-dioxane and THF, which were purchased from Carl Roth (HPLC grade, Karlsruhe, Germany).

NMR measurements were carried out on a 300 MHz Bruker NMR spectrometer (Karlsruhe, Germany). The solvent used is specified for each spectrum shown. The spectra were referenced to the residual solvent signal.

Size exclusion chromatography in tetrahydrofuran (THF-SEC) was performed on an Agilent 1260 system equipped with a G1330B pump, a PSS TC6001 oven at 30 °C, a G1362A refractive index detector, and a G1315D UV detector at 365 nm. THF was used as eluent at a flow rate of 1 mL min^{-1} on three PSS SDV guard columns (100/1000/100 000 Å). The system was calibrated with polystyrene, poly(methyl methacrylate), polyethylene glycol, and polyisoprene standards from PSS (Mainz, Germany) with a molecular weight range of 200–2 000 000 g mol^{-1}.

SPA monomer synthesis:

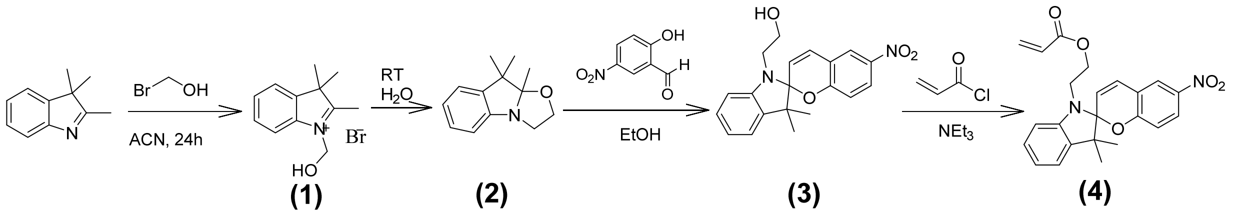

Figure S1. Synthetic route for 2-(3',3'-Dimethyl-6-nitrospiro[chromene-2,2'-indolin]-1'-yl)ethyl Acrylate (SPA)

(1) *Synthesis of 1-(2-Hydroxyethyl)-2,3,3-trimethyl-3H-indolium Bromide*

A mixture of 2,3,3-trimethyl-3H-indol (2.72 mL, 16 mmol) and 2-bromoethanol (1.48 mL, 20 mmol, 1.25 eq) in acetonitrile (20 mL) was reflux at 100 °C under Argon for 24 hours. The solvent was removed and resuspended in 25 mL hexane. The collected solid was recrystallized from chloroform and directly used in the following step.

(2) *Synthesis of 9,9,9 a-Trimethyl-2,3,9,9a-tetrahydro-oxazolo[2,3-a]indole*

1-(2-Hydroxyethyl)-2,3,3-trimethyl-3H-indolium bromide **1** was dissolved in aqueous solution mixed with KOH (0.66 g, 12 mmol). The mixture turned from pink to dark yellow within 10 min at room temperature. The mixture was then extracted with diethylether (3 × 20 mL), dried over $MgSO_4$, filtered, and the solvent removed under reduced pressure. The yellow oil (0.8549 g, 33.5%) was analyzed *via* ^{1}H NMR:

^{1}H NMR (300 MHz, $CDCl_3$): δ = 1.89 (s, 3H, CH_3), 1.31 (s, 3H, CH_3), 1.42 (s, 3H, CH_3), 3.44–3.86 (m, 4H, 2 × CH_2), 6.76 (d, 1H, CH-arom.), 6.93 (t, 1H, CH-arom.), 7.14 (m, 2H) ppm.

(3) *Synthesis of 2-(3',3'-Dimethyl-6-nitro-3'H-spiro[chromene-2,2'-indol]-1'-yl)-ethanol*

9,9,9a-trimethyl-2,3,9,9a-tetrahydro-oxazolo[2,3-*a*]indole **2** (0.85 g, 4,2 mmol, 1 eq) and 2-hydroxy-5-nitrobenzaldehyde (1.054 g, 6.3 mmol, 1.5 eq) were mixed in 10 mL of ethanol and heated under reflux for 3 h. After cooling to room temperature, the remaining solution was filtered and washed with ethanol. The red crystals (0.6319 g, 33%) were analyzed *using* ^{1}H NMR:

^{1}H NMR (300 MHz, DMSO-d_6): δ = 1.10 (s, 3H, CH_3), 1.20 (s, 3H, CH_3), 3.19 (dq, 2H, CH_2), 3.44

(m, 2H, CH_2), 4.72 (t, 1H, OH), 6.01 (d, 1H, CH-arom.), 6.64 (d, 1H, CH-arom.), 6.78 (t, 1H, CH-arom.), 6.87 (d, 1H, CH-arom.), 7.11 (m, 3H, 3 × CH-arom.), 8.00 (dd, 1H, CH-arom.), 8.21 (d, 1H, CH-arom) ppm.

*(4) Synthesis of 2-(3',3'-Dimethyl-6-nitrospiro[chromene-2,2'-indolin]-1'-yl)ethyl Acrylate (*SPA)2-(3′,3′-Dimethyl-6-nitro-3′H-spiro[chromene-2,2′-indol]-1′-yl)-ethanol **3** (0.5 g, 1.42 mmol) and trimethylamine (0.197 mL, 1.42 mmol) were dissolved under Argon in dichloromethane, and then the mixture was cooled to −35 °C. A solution of acryloyl chloride in dichloromethane was dropped slowly and the reaction mixture was heated up to room temperature overnight. The solution was extracted with saturated $NaHCO_3$ (2 × 20 mL) and water (2 × 20 mL), and the organic phases were combined, dried over $MgSO_4$, filtered, and evacuated under reduced pressure. The product was purified using column chromatography with chloroform to yield 107.6 mg (0.265 mmol, 18.6%).

^{1}H NMR (300 MHz, DMSO-d_6): δ = 1.06 (s, 3H, CH_3), 1.19 (s, 3H, CH_3), 3.36–3.51 (m, 2 × 1H, CH_2), 4.16–4.35 (m, 2 × 1H, CH_2), 5.90 (d, 1H, CH_2) 5.96 (d, 1H, CH-arom.), 6.09 (dd, 1H, CH), 6.26 (d, 1H, CH_2), 6.72 (d, 1H, CH-arom.), 6.80 (t, 1H, CH-arom.), 6.85 (d, 1H, CH-arom.), 7.12 (m, 2H, 2 × CH-arom.), 7.20 (d, 1H, CH-arom.), 7.99 (dd, 1H, CH-arom.), 8.21 (d, 1H, CH-arom.) ppm.

Synthesis of P(TEGA-*co*-SPA)

SPA (20mol % SPA), *triethylene glycol acrylate (*TEGA*)* (1043 mg, 5.5 mmol), and azobisisobutyronitrile (2.3 mg initiator M/I = 400) were dissolved in 3 mL dioxane in a microwave vial. The solution was purged with argon for 30 min and heated to 110 °C for 48 h. The resulting red solution was precipitated twice into cold diethylether, resulting in a red powder (600.6 mg, 49.7% yield). The copolymers were analyzed *using* SEC and ^{1}H NMR in $CDCl_3$.

^{1}H-NMR (300 MHz, $CDCl_3$): δ = 3.3 (s, 3H, CH3, TEGA), and 8 (s, 1H, CH arom., SPA) ppm.

SEC (THF): Mn 27000, and PDI: 2.

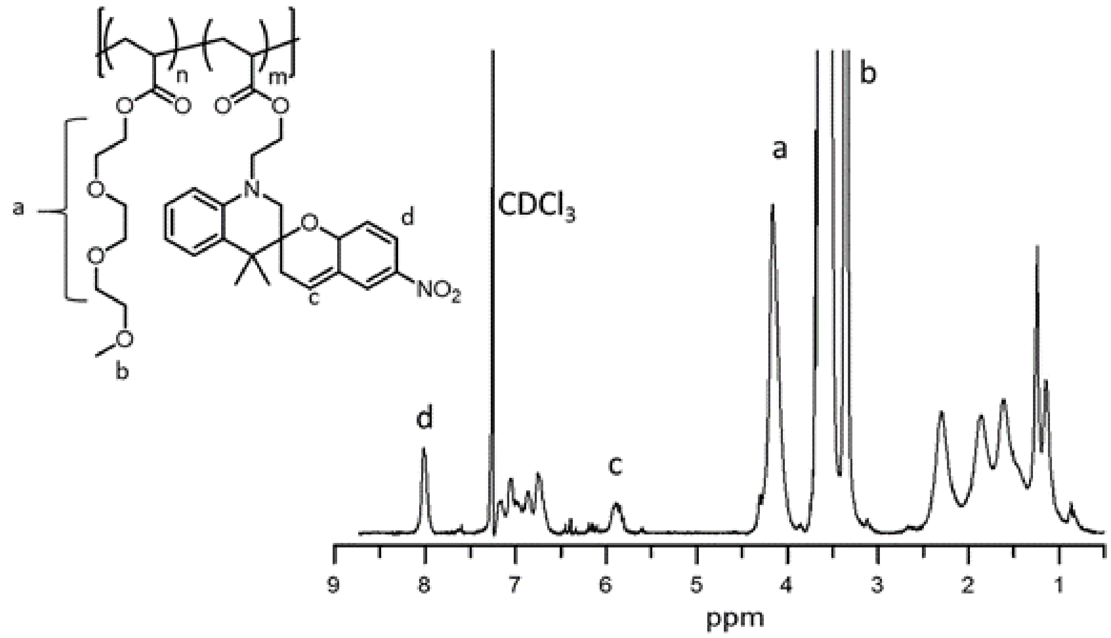

Figure S2. 1H-NMR of P(TEGA-co-SPA) copolymer in CDCl3.

Quartz crystals in AT-cut with fundamental resonance frequency of ~5 MHz (shear mode) were used. Since the shear oscillation is sensitive to temperature induced changes of dynamic viscosity of the solvent water, experimental data was normalized. Prior to experiments, a reference scan was conducted for each quartz crystal employed over the temperature interval of 20 - 50 °C using pure water. The observed resonant frequency shift $\Delta f_3/3$ and dissipation shift ΔD_3 of the water background is shown in Figure S2. The temperature dependencies of resonance parameters f and Γ were fitted with polynomial functions for correction of the dynamic viscosity of water.

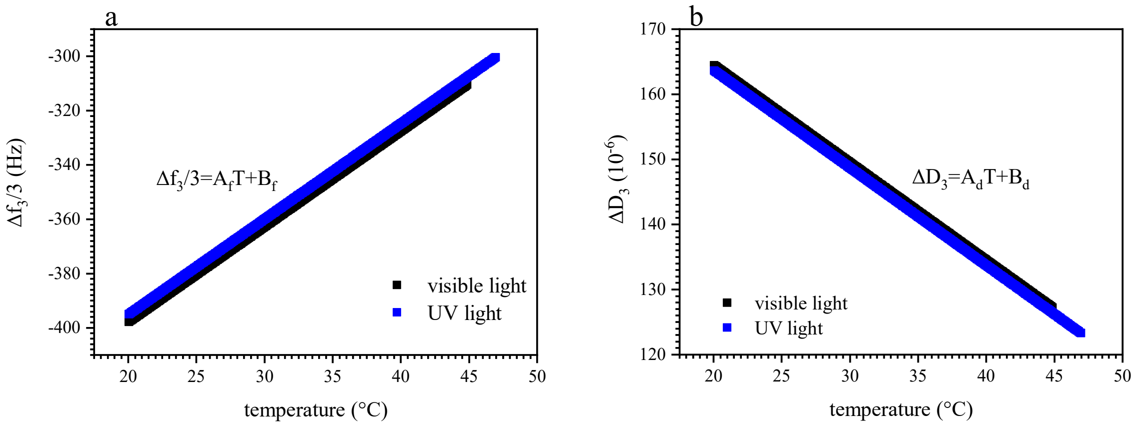

Figure S3. QCM-D water background used for data correction.

Table S1. Fit parameters for the temperature correction as indicated in Figure S3.

**Irradiation**	A_f	B_f	A_d	B_d
**Visible light**	**3.52**	**-468.4**	**-1.5**	**194.5**
**UV light**	**3.52**	**-465.6**	**-1.5**	**193.7**

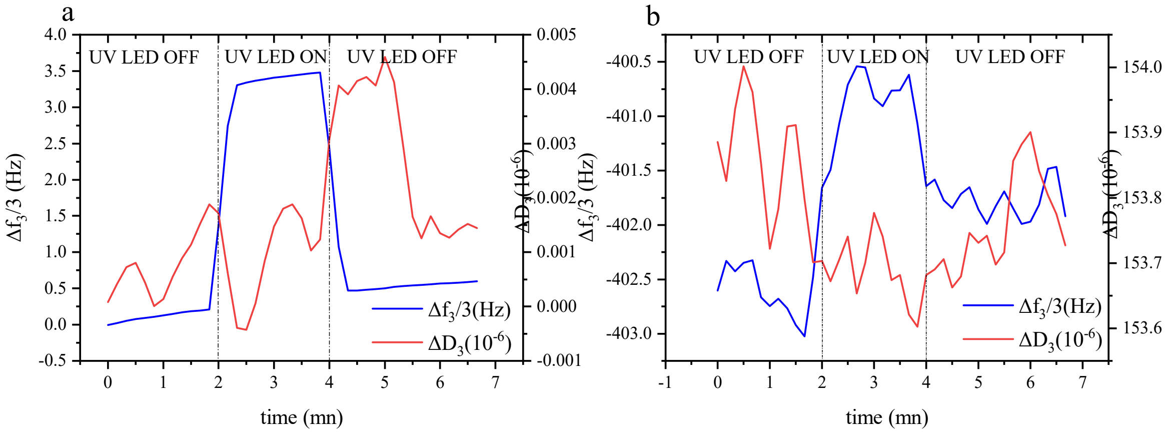

Figure S4. Effect of UV illumination on bare (a) and water-wet sensor (b).

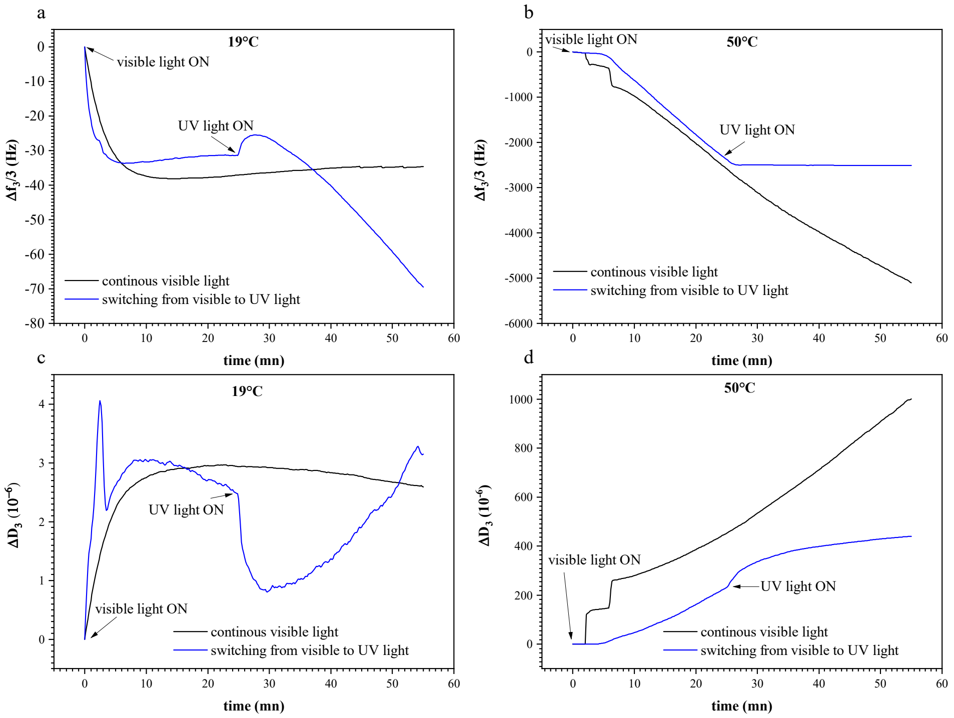

Figure S5. Effect of continuous visible and UV light irradiation on the resonance frequency shift $\Delta f_3/3$ (a,b) and dissipation shift ΔD3 (c,d) on the copolymer liquid film at 19 and 50°C, respectively.

References

(1) Grimm, O.; Schacher, F. H. Dual Stimuli-Responsive P(NIPAAm-*co*-SPA) Copolymers: Synthesis and Response in Solution and in Films. *Polymers.* **2018**, *10* (6). https://doi.org/10.3390/polym10060645.
